# Cholesterol in hematological malignancies: metabolism, mechanisms, and clinical implications

**DOI:** 10.3389/fimmu.2026.1947729

**Published:** 2026-09-21

**Authors:** Yaqin Zhang, Mengbo Yang, Siyuan Yan, Ruiyang Li, Yuanjun Zheng, Donglei Zhang, Hongyuan Shang, Fang Wei

**Affiliations:** 1 The First Clinical Medical College of Shanxi Medical University, Taiyuan, Shanxi, China; 2 Precision Medicine Laboratory for Chronic Non-Communicable Diseases of Shandong Province, Institute of Precision Medicine, Jining Medical University, Jining, China; 3 Department of Hematology, The First Hospital of Shanxi Medical University, Taiyuan, Shanxi, China; 4 Department of Ophthalmology, The First Hospital of Shanxi Medical University, Taiyuan, Shanxi, China; 5 College of Pharmacy, Shanxi Medical University, Taiyuan, China

**Keywords:** cholesterol, clinical significance, hematological malignancies, hypocholesterolemia, metabolic reprogramming, molecular mechanisms

## Abstract

Cholesterol is an amphiphilic molecule that is essential for maintaining membrane structure and integrity, serves as a precursor for steroid hormone synthesis, and plays a critical role in metabolic homeostasis and physiological function. In recent years, dysregulated cholesterol metabolism has emerged as a major focus of research in the field of hematological malignancies. A growing body of clinical evidence indicates that hypocholesterolemia is a common concomitant condition in various hematological malignancies and is closely associated with disease onset, progression, therapeutic response, and clinical outcomes. The mechanisms underlying tumor-associated hypocholesterolemia are complex and likely involve dysregulation of lipoprotein metabolism, liver dysfunction caused by systemic inflammatory responses, and abnormal cholesterol uptake and efflux by tumor cells, which collectively contribute to the exacerbation of hypocholesterolemia. In turn, this metabolic signature not only promotes tumor cell proliferation and enhances the potential for therapy resistance, but also reprograms the immune microenvironment by inducing T cell dysfunction and M2-like macrophage polarization. Emerging therapeutic strategies targeting cholesterol metabolism have shown promise in preclinical models. By providing a theoretical foundation, these strategies offer potential avenues for improving the early diagnosis and risk stratification of hematological malignancies and for developing innovative treatment regimens. In this review, we outline the biosynthesis, uptake, excretion, transformation, and regulatory networks of cholesterol; describe the specific alterations in cholesterol levels across different subtypes of hematological malignancies; elucidate the molecular mechanisms underlying hypocholesterolemia; and explore its effects on tumor cell biology and the immune microenvironment, as well as its clinical significance, with the aim of providing new perspectives for related treatment strategies.

## 1 Introduction

Tumor cells undergo metabolic reprogramming to meet the biosynthetic and bioenergetic demands of carcinogenesis. Cholesterol, a major component of the cell membrane, plays a critical role in maintaining membrane integrity and fluidity, facilitating the formation of membrane microdomains, and supporting cellular structure and proliferation (1). In addition, it not only serves as a precursor for the synthesis of bile acids, vitamin D, and steroid hormones (2), but also acts as a covalent protein modifier, thereby contributing to a wide range of biological processes. Intracellular cholesterol metabolic homeostasis is maintained by a complex regulatory network governing biosynthesis, uptake, efflux, conversion, esterification, and trafficking (3). Previous studies have shown that alterations in lipid metabolism are associated with the development of certain cancers (4). Evidence indicates that serum cholesterol levels begin to decline up to six years prior to the clinical detection of colon cancer and chronic myeloid leukemia (5), suggesting that cholesterol levels may serve as a predictive marker for early carcinogenesis.

Over the past decade, an increasing number of studies have demonstrated that dysregulated lipid metabolism, particularly alterations in cholesterol homeostasis, is closely associated with the initiation, progression, and prognosis of hematological malignancies. In-depth investigation of the mechanistic role of cholesterol in these tumors not only promises to elucidate novel pathogenic mechanisms and provide new biomarkers for early diagnosis and risk stratification, but also establishes a theoretical foundation for the development of innovative therapeutic strategies. These may include targeted agents directed against key nodes within the cholesterol metabolic pathway. Such investigations hold substantial clinical significance and considerable translational potential.

## 2 Cholesterol: structure, function, and homeostatic regulation

The mechanisms underlying cholesterol homeostasis are complex and are regulated through the integration of four key metabolic processes: synthesis, absorption, transformation, and clearance (**Figure 1**). This coordinated metabolic cycle ensures that cholesterol fulfils its essential roles in membrane structure, signal transduction, and systemic homeostasis.

**Figure 1:**
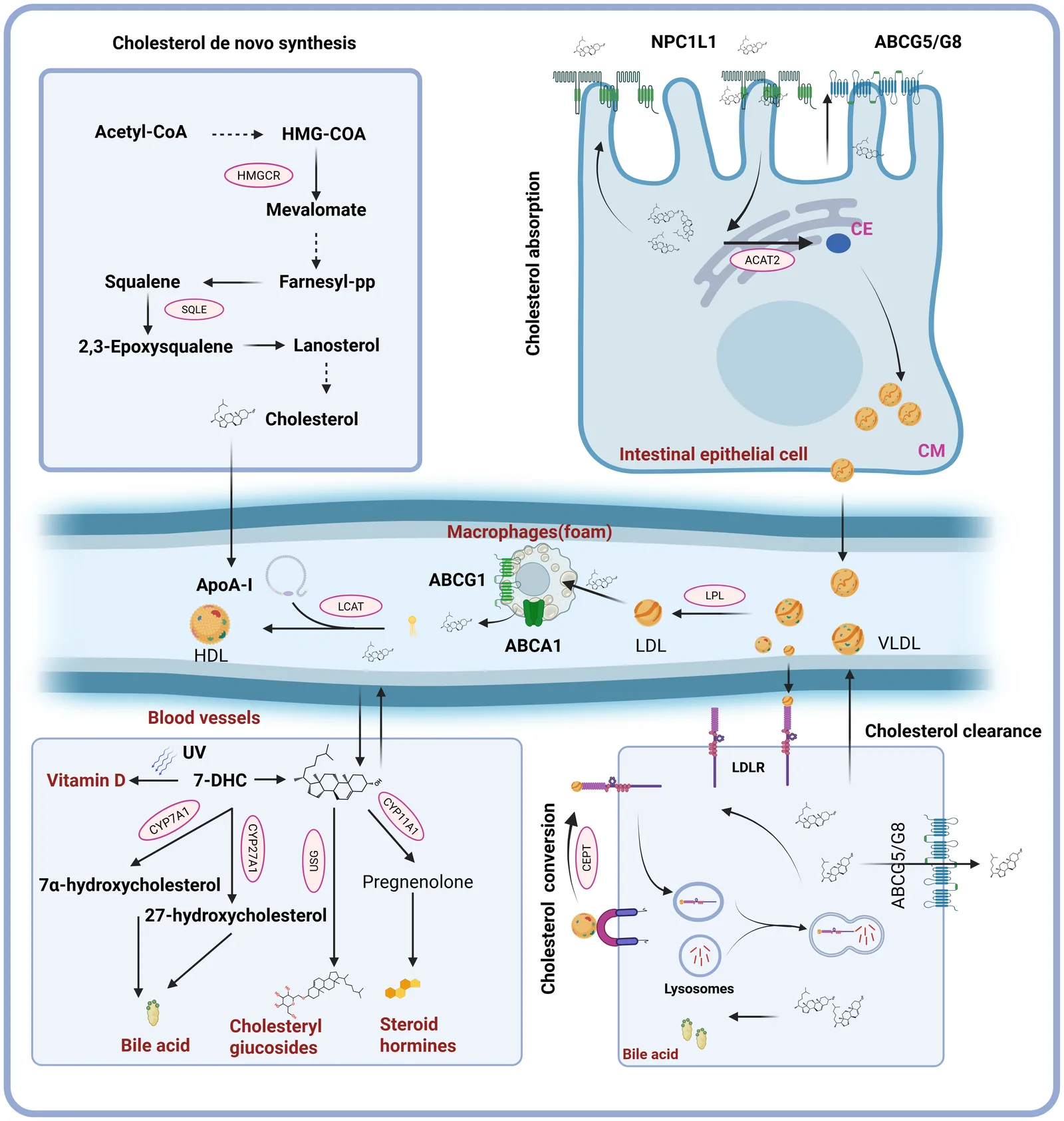
An overview of cholesterol homeostasis (Created in BioRender). In intestinal epithelial cells, cholesterol is taken up via NPC1L1 and effluxed by ABCG5/G8, while ACAT2 converts it into cholesteryl esters (CE) for chylomicron (CM) formation. *De novo* synthesis begins with acetyl-CoA and involves HMGCR and SQLE. In the circulation, lipoproteins (VLDL, LDL, HDL) mediate cholesterol transport; LDLR facilitates clearance, while ABCA1 and ABCG1 promote cholesterol efflux to HDL. Macrophages may become foam cells upon cholesterol accumulation. Cholesterol is also converted into bile acids (via CYP7A1), steroid hormones, vitamin D, and oxysterols such as 7α-hydroxycholesterol and 27-hydroxycholesterol. Lysosomal trafficking and glucosylation (ABCG5/G8) further regulate sterol distribution.

### 2.1 Classification and structure of cholesterol

Cholesterol (molecular formula: C27H46O) is a cyclopentane-polyhydrophenanthrene derivative. It consists of three six-membered rings (A, B, and C) fused to a five-membered ring (D). The molecule contains a 3β-hydroxyl group at the C3 position and a double bond between the C5 and C6 positions, with an eight-carbon alkyl side chain attached at the C17 position. It is an amphiphilic molecule that serves as a key structural component of cell membranes and as a precursor for steroid hormones (6). In plasma, cholesterol exists as free cholesterol (FC, approximately 30%) and cholesteryl esters (CE, approximately 70%), which are formed through esterification catalysed by Lecithin-Cholesterol Acyltransferase (LCAT). These esters are hydrophobic and reside within the core of lipoproteins (2). Based on their lipoprotein carriers, cholesterol is classified into chylomicrons (CM), very-low-density lipoproteins (VLDL), intermediate-density lipoproteins (IDL), low-density lipoproteins (LDL), and high-density lipoproteins (HDL) (4, 7).

### 2.2 Cholesterol biosynthesis

Cholesterol biosynthesis occurs in most mammalian cells, with endogenous synthesis in the liver and dietary absorption representing the two principal sources of cholesterol in the human body (8). Under physiological conditions, endogenous synthesis predominantly takes place in the liver, where acetyl-CoA is converted into cholesterol through nearly 30 enzymatic reactions, including the mevalonate (MVA) pathway and subsequent squalene biosynthesis (9, 10). Among the enzymes involved, 3-hydroxy-3-methylglutaryl-coenzyme A (HMG-CoA) reductase (HMGCR) and squalene epoxidase (SQLE) are key rate-limiting enzymes that catalyse the reduction of HMG-CoA to MVA and the epoxidation of squalene to 2,3-epoxysqualene, respectively (11).

### 2.3 Cholesterol absorption

In addition to biosynthesis, dietary absorption and subsequent uptake of cholesterol from the circulation play crucial roles in maintaining cholesterol homeostasis. The exogenous absorption of cholesterol is a multi-step process coordinated by multiple proteins. Cholesterol derived from food and bile, following hydrolysis and micellisation, is absorbed by intestinal epithelial cells primarily via Niemann-Pick C1-Like 1 (NPC1L1). Through a series of processes, free cholesterol taken up by NPC1L1 is esterified by cholesterol acyltransferase 2 (ACAT2) in the endoplasmic reticulum and subsequently transported into the circulation in the form of chylomicrons, ultimately being assimilated by the liver (12, 13). Conversely, unesterified free cholesterol is actively transported into the intestinal lumen by the heterodimeric ATP-binding cassette (ABC) transporters G5/G8 (ABCG5/G8), thereby limiting net absorption. Furthermore, under conditions of low cholesterol, NPC1L1 can recycle back to the cell membrane to sustain cholesterol uptake (14). Ultimately, cholesterol is taken up and utilised by the liver and peripheral tissues through receptor-mediated mechanisms, primarily involving the low-density lipoprotein receptor (LDLR) and scavenger receptor class B type I (SR-BI), thereby serving as a source of exogenous cholesterol for the body (15). Key proteins at each stage, including NPC1L1, LDLR, and SR-BI, play central roles in maintaining cholesterol homeostasis (1, 16). While most mammalian cells obtain cholesterol via endogenous or exogenous uptake (17), certain specialised cells, such as intestinal epithelial cells and hepatocytes, have additional cholesterol sources, including direct absorption from the intestinal tract and bile via the cholesterol transporter NPC1L1 (18).

### 2.4 Cholesterol conversion and clearance

As a key metabolic precursor, cholesterol can be converted into a variety of bioactive molecules with important physiological functions across multiple tissues. In the liver, cholesterol is converted into bile acids predominantly via the classical pathway (accounting for > 90% of total conversion). This pathway is initiated by the rate-limiting enzyme cytochrome P450 family 7 subfamily A member 1 (CYP7A1), which catalyses the conversion of cholesterol to 7α-hydroxycholesterol; this intermediate is subsequently converted to C4, ultimately leading to the synthesis of cholic acid and chenodeoxycholic acid (19). When the classical pathway is impaired or under metabolic stress, an alternative pathway initiated by CYP27A1 generates 27-hydroxycholesterol, which is further catalysed by CYP7B1 to supplement the production of chenodeoxycholic acid (20). Intracellularly, cholesterol can be converted into cholesterol glucoside by UDP-glucose:sterol glucosyltransferase, thereby contributing to membrane stability and immune modulation (21). In the skin, 7-dehydrocholesterol is converted into active vitamin D3 following UVB exposure and subsequent two-step hydroxylation in the liver and kidneys (22). In the adrenal cortex, cholesterol is converted into pregnenolone by CYP11A1 and is subsequently synthesised into steroid hormones.

Systemic clearance of cholesterol is primarily achieved through two pathways: HDL-mediated reverse cholesterol transport and LDL-mediated hepatic uptake (23). During reverse cholesterol transport, nascent HDL is assembled from apolipoprotein A-I (ApoA-I) and phospholipids (24). It acquires free cholesterol from peripheral cells, such as macrophages, via ABCA1/ABCG1; this cholesterol is then esterified by LCAT to form mature HDL particles (25). These cholesteryl esters are either selectively taken up by hepatocytes via SR-BI or transferred to apolipoprotein B-containing lipoproteins (such as LDL and VLDL) via Cholesteryl Ester Transfer Protein (CETP), and are ultimately cleared through the LDLR-mediated hepatic pathway. In the LDL clearance pathway, LDL particles bind to LDLR on the surface of hepatocytes via apolipoprotein ApoB-100 (26). Following internalisation, cholesteryl esters are hydrolysed into free cholesterol, while LDLR is recycled back to the cell membrane to maintain uptake capacity.

### 2.5 Biological functions and signaling-regulatory roles of cholesterol

Cholesterol is a multifunctional molecule essential for maintaining membrane integrity and fluidity, accounting for 20-30% of membrane lipids. It is embedded within the phospholipid bilayer, where it buffers membrane phase transitions, preventing excessive fluidity at high temperatures and the formation of rigid, gel-like structures at low temperatures (27). In addition to this structural role, cholesterol forms lipid raft microdomains in association with sphingolipids, which serve as platforms for signal transduction, receptor activation, and immunocontact formation (28, 29). Cholesterol serves as the universal precursor for all steroid hormones (30). Through the action of the rate-limiting enzyme CYP11A1 (cholesterol side-chain cleavage enzyme) (31), cholesterol is converted into pregnenolone, which is subsequently synthesised into mineralocorticoids, glucocorticoids, and sex hormones, thereby regulating electrolyte balance, stress responses, and reproductive physiology (30). Furthermore, cholesterol acts as a vital modulator of cellular signaling pathways. It regulates ion channel functions through specific mechanisms (32), and directly binds to Patched-1 (PTCH1) and Smoothened (SMO) to modulate Hedgehog signaling and precisely control developmental and homeostatic processes (33). The dysfunction of cholesterol-dependent signaling networks facilitates the progression of hematologic malignancies. Cholesterol preserves the structural integrity of lipid rafts to sustain normal PI3K/Akt signaling transduction (34, 35). Its deficiency induces adaptive survival programs in tumor cells and ultimately leads to chemotherapy resistance. Upon activation, SMO signals to downstream GLI transcription factors. This signaling axis maintains the self-renewal of leukemia stem cells (36) and exerts oncogenic functions in multiple myeloma and lymphoma (37). Cholesterol metabolism modulates TCR complex assembly to alleviate T cell exhaustion and cross-regulates the mTORC1 signaling pathway (38–40). LRP8-mediated cholesterol metabolism further controls mTORC1 activity and tumor cell apoptosis in multiple myeloma (41). In conclusion, cholesterol serves as a core mediator linking metabolic reprogramming and oncogenic signaling. It plays a crucial pathological and clinical role in the progression, immune escape and treatment resistance of hematologic malignancies (**Figure 2**).

**Figure 2:**
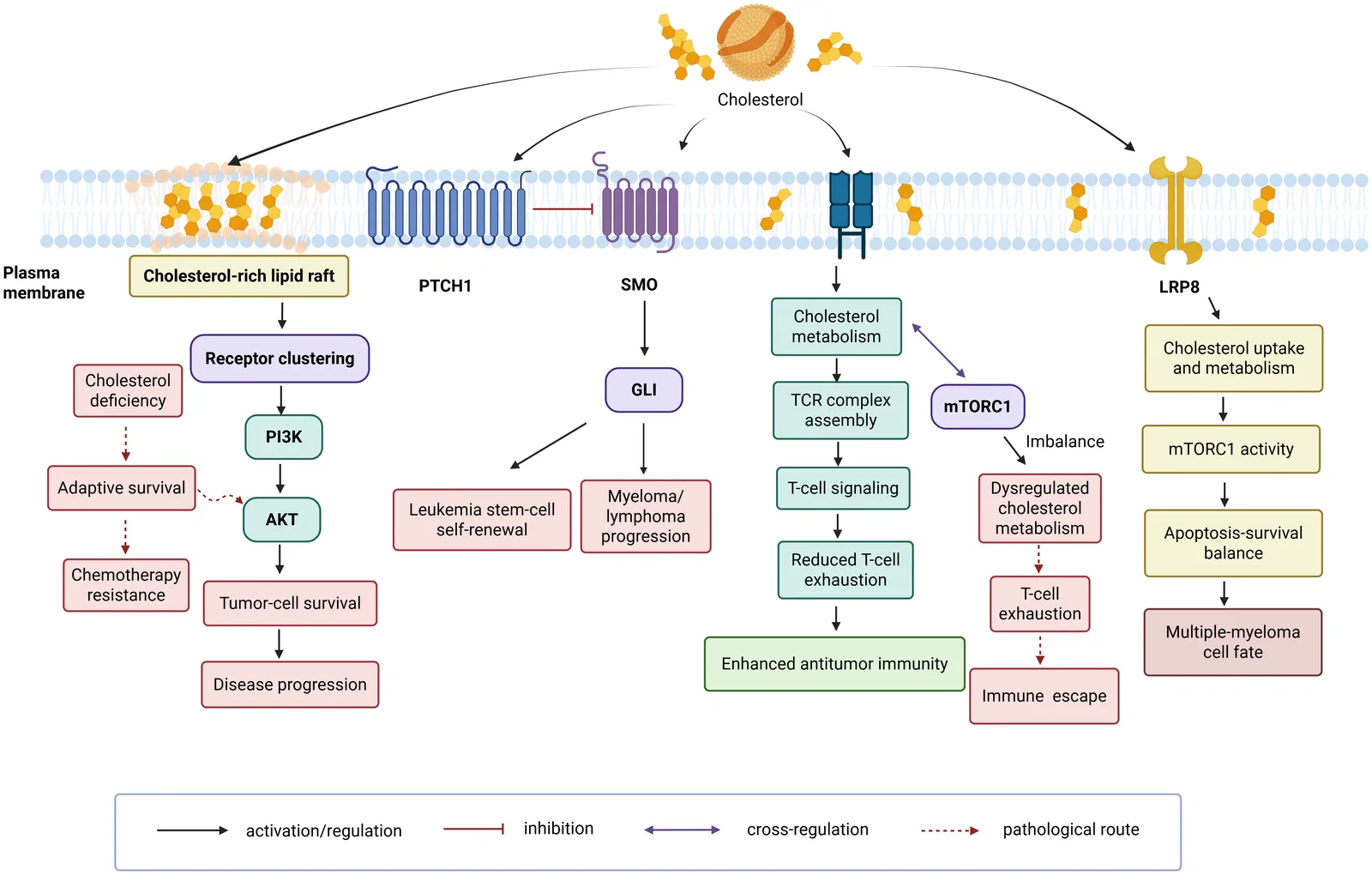
Cholesterol-regulated signal transduction axes in hematological malignancies (Created in BioRender). Cholesterol is embedded in plasma membrane lipid rafts, where it regulates receptor clustering (e.g., PTCH1, SMO, LRP8) and modulates key downstream signaling cascades, including the Hedgehog/GLI, PI3K/Akt, TCR, and LRP8/mTORC1 axes. Under physiological conditions, these pathways coordinate normal T-cell activation, survival homeostasis, and antitumor immunity. However, cholesterol deficiency or metabolic reprogramming disrupts these networks, driving leukemia stem cell self-renewal, promoting myeloma/lymphoma progression, inducing T-cell exhaustion and immune evasion, and ultimately conferring chemotherapy resistance and adverse clinical outcomes. Solid arrows, T-bars, and dashed lines indicate activation, inhibition, and cross-regulation, respectively.

### 2.6 Regulation of cholesterol homeostasis

Cholesterol homeostasis is tightly regulated by two families of transcription factors: sterol regulatory element-binding proteins (SREBPs) (42) and liver X receptors (LXRα and β isoforms) (43, 44). Sterol regulatory element-binding protein 2 (SREBP2) regulates cholesterol uptake by inducing the expression of the LDLR (2). In contrast, LXRs promote cholesterol efflux and facilitate its transport back to the liver via reverse cholesterol transport for excretion (45). Dysregulation of cholesterol homeostasis is a characteristic feature of cancer cells. Owing to their markedly increased proliferation rates compared with normal cells, cancer cells require higher levels of cholesterol for membrane biogenesis and signal transduction (46, 47). Intracellular cholesterol levels are regulated by these transcription factors. Cholesterol depletion activates SREBPs, leading to upregulation of genes such as HMGCR and LDLR, thereby promoting cholesterol synthesis and uptake. Conversely, when cholesterol levels are elevated, LXRs are activated, stimulating the expression of genes involved in cholesterol efflux and excretion, including ABCA1 and ABCG1, and facilitating transport back to the liver in the form of HDL complexes (48, 49). This establishes a finely tuned feedback regulatory circuit (50). Tumor cells exploit this regulatory system to meet the metabolic demands of malignant proliferation.

In hematologic malignancies, SREBP2 is activated in AML, DLBCL, and MM, upregulating LDLR/HMGCR/SQLE to enhance cholesterol uptake/synthesis (42, 51, 52). In contrast, LXRs are suppressed, reducing ABCA1/ABCG1 efflux and promoting cholesterol accumulation for clonal growth (43–45), though LXR may induce apoptosis in MM (41). This SREBP-activated/LXR-repressed axis drives metabolic reprogramming. In T cells, SREBP supports lipid raft integrity and TCR signaling; in CLL, impaired SREBP1/2 activation downregulates LDLR expression and reduces cholesterol uptake, promoting T-cell exhaustion and compromising the efficacy of immune checkpoint inhibitors—a defect further exacerbated by hypocholesterolemia (53, 54). In macrophages, SREBP2 drives M1 polarization, but low cholesterol favors M2-like TAMs with IL-10 (55, 56). LXR regulates macrophages via efflux/anti-inflammation, varying by model. SREBP supports HSC self-renewal, LXRs regulate quiescence; dysregulation aids preleukemic evolution (47). This cell-type-specific regulation forms a metabolic-immune network with therapeutic potential.

In sharp contrast to normal hematopoietic cells—where SREBP2 and LXRs operate in a balanced feedback loop to maintain physiological cholesterol levels—malignant cells in hematologic neoplasms reprogram this regulatory circuitry toward a “dual-assurance” strategy. This is characterized by sustained SREBP2-driven upregulation of LDLR/HMGCR/SQLE to maximize cholesterol uptake and *de novo* synthesis, coupled with LXR-mediated suppression of ABCA1/ABCG1 to minimize cholesterol efflux. Such a polarized metabolic adaptation not only ensures sufficient cholesterol supply for rapid membrane biosynthesis and oncogenic signaling, but also distinguishes tumor cells from their normal counterparts, providing a mechanistic basis for the hypocholesterolemia observed in patients.

## 3 Cholesterol levels in hematological malignancies

Cholesterol is indispensable for cancer cell proliferation and performs several critical biological functions. Studies have shown that patients with lung cancer (57, 58), gastric cancer (59), thyroid cancer (60), breast cancer (61), ovarian cancer (62), and prostate cancer (63) often exhibit hypocholesterolemia, which may result from increased demand for cholesteryl ester uptake. In addition to solid tumors, reduced cholesterol levels have also been observed in patients with hematological malignancies (64), including chronic lymphocytic leukemia (65), acute lymphoblastic leukemia (66), lymphoma (67), and multiple myeloma (41) (**Table 1**).

**Table 1:** Cholesterol levels in hematological malignancies and clinical correlation.

Tumor category	Specific subtype	TC level	LDL-C level	HDL-C level	Clinical correlation	Reference
Leukemia	Acute promyelocytic Leukemia (APL)	↓	↓	↓	LDL-C level is a critical prognostic indicator.	(69)
	Chronic Lymphocytic Leukemia (CLL)	↓	↓OR↑	↓	Synchronous decrease at diagnosis is a powerful independent adverse prognostic marker.	(126)
Lymphoma	Diffuse Large B-cell Lymphoma (DLBCL)	↓	↓	↓	High HDL-C levels are associated with a reduced risk of DLBCL.	(79)
	Hodgkin Lymphoma (HL)	——	——	↓	HDL-C level can serve as a potential biomarker of disease activity.	(81)
Multiple Myeloma(MM)	Newly Diagnosed Multiple Myeloma (NDMM)	↓	↓	↓	Sterol levels may dynamically reflect disease activity in MM.	(85)

### 3.1 Leukemia

Acute myeloid leukemia (AML) comprises a heterogeneous group of malignancies characterised by the abnormal proliferation of hematopoietic progenitor cells and impaired normal hematopoiesis; if left untreated, patients often die within weeks due to infection, bleeding, or organ failure (68). Current evidence regarding serum cholesterol levels in patients with leukemia remains inconsistent. Some studies report that total cholesterol, high-density lipoprotein cholesterol (HDL-C), and low-density lipoprotein cholesterol (LDL-C) levels are significantly reduced in patients with acute promyelocytic leukemia (APL), chronic lymphocytic leukemia (CLL), and acute non-lymphocytic leukemia (ANLL) (65, 69). In contrast, other studies have found that hypercholesterolemia is relatively common in patients with CLL, with 75% exhibiting elevated LDL-C levels (70), while additional reports indicate that only HDL-C levels are reduced, whereas total cholesterol, LDL-C, and triglyceride levels remain stable or show slight increases (71). These discrepancies may reflect differences in cellular differentiation status and experimental methodologies. Nevertheless, it is widely accepted that leukemic cells exhibit increased cholesterol utilisation (72, 73) and upregulated synthesis (74). Notably, elevated total cholesterol levels have been observed in patients demonstrating a therapeutic response across different subtypes of leukemia (75).

### 3.2 Lymphoma

Lymphoma encompasses a heterogeneous group of malignant tumors originating from lymphocytes (B cells, T cells, or NK cells), characterised by clonal proliferation, most commonly occurring in lymph nodes or lymphoid tissues. It is primarily classified into two major categories: Hodgkin lymphoma and non-Hodgkin lymphoma.

Multiple studies have shown that patients with lymphoma exhibit dysregulated cholesterol metabolism, with levels of total cholesterol (TC), LDL-C, and HDL-C significantly lower than those in healthy individuals (76). Notably, TC, triglycerides (TG), and ApoA-I are negatively correlated with non-Hodgkin lymphoma (NHL) (77). Clinical evidence further supports this observation: a large-scale cohort study demonstrated that cholesterol levels in affected individuals began to decline four years prior to diagnosis, and this change was independent of treatment, suggesting that it may represent an early disease-related alteration (76, 78). Furthermore, across various lymphoma subtypes, similar reductions in TC, HDL-C, and LDL-C levels have been observed five to ten years before diagnosis (76). Among these markers, higher HDL-C levels are associated with a reduced risk of diffuse large B-cell lymphoma (DLBCL) (79). This finding is supported by a Japanese study showing that patients with malignant lymphoma (ML) and adult T-cell leukemia/lymphoma (ATLL) had lower HDL-C and LDL-C levels than controls, and that HDL-C was negatively correlated with soluble interleukin-2 receptor (sIL-2R). Importantly, patients with the lowest HDL-C levels (<0.52 mmol/L) had a poorer prognosis, with a hazard ratio of 5.73 (80, 81). However, in patients with Hodgkin’s lymphoma, HDL-C levels were significantly lower than those in healthy controls both at diagnosis and during the active phase of the disease, whereas TC and LDL-C did not show a significant decrease (81).

### 3.3 Multiple myeloma

Multiple myeloma (MM) is a malignant tumor originating from plasma cells in the bone marrow. It is characterised by the abnormal proliferation of monoclonal plasma cells and is often accompanied by hypercalcemia, renal impairment, anemia, and bone destruction. Multiple retrospective clinical analyses have demonstrated significantly reduced serum cholesterol levels in patients with MM, with this parameter correlating with disease stage, tumor burden, treatment response, and prognosis (82–84). TC and LDL-C are inversely associated with MM. Consistent with this, Kuliszkiewicz-Janus et al. reported significant reductions in HDL-C, LDL-C, and TC levels in patients with newly diagnosed MM. Notably, these cholesterol levels appear to return to normal during disease remission, suggesting that cholesterol levels may dynamically reflect disease activity (85). Similar findings have been reported in broader MM cohorts; compared with healthy individuals, patients with MM consistently exhibit significantly lower levels of TC, HDL-C, and LDL-C. Furthermore, this reduction in cholesterol levels is associated with disease severity, with levels declining further as tumor stage advances (83). In addition to disease stage and activity, cholesterol profiles differ significantly according to MM immunotype. Specifically, among patients with different immunotypes, the levels of TC, LDL-C, HDL-C, Apo A-I, and apolipoprotein B (Apo B) exhibit clear hierarchical differences: cholesterol-related indicators in IgA-type MM are significantly lower than those in IgG-type patients; indicators in IgG-type patients are significantly lower than those in light chain-type patients; and indicators in double clone-type patients are also significantly lower than those in light chain-type patients (86).

## 4 The mechanism of hypocholesterolemia in hematological malignancies

Hypocholesterolemia in patients with hematological malignancies represents a multifactorial and complex pathophysiological state, with its core mechanism rooted in the interaction between tumor cell “metabolic hijacking” and disruption of host systemic metabolic homeostasis. Specifically, malignant tumor cells exhibit increased cholesterol consumption by upregulating both uptake and endogenous synthesis pathways. Simultaneously, tumor-associated systemic inflammatory responses suppress hepatic lipid metabolism, leading to impaired cholesterol synthesis and secretion.

Superimposed on this metabolic reprogramming are additional factors that further exacerbate the decline in cholesterol levels, including malnutrition, which is common in advanced disease stages, and the direct adverse effects of antineoplastic therapies on hepatic function and intestinal absorption. Collectively, these factors constitute the integrated pathological basis for hypocholesterolemia (**Figure 3**).

**Figure 3:**
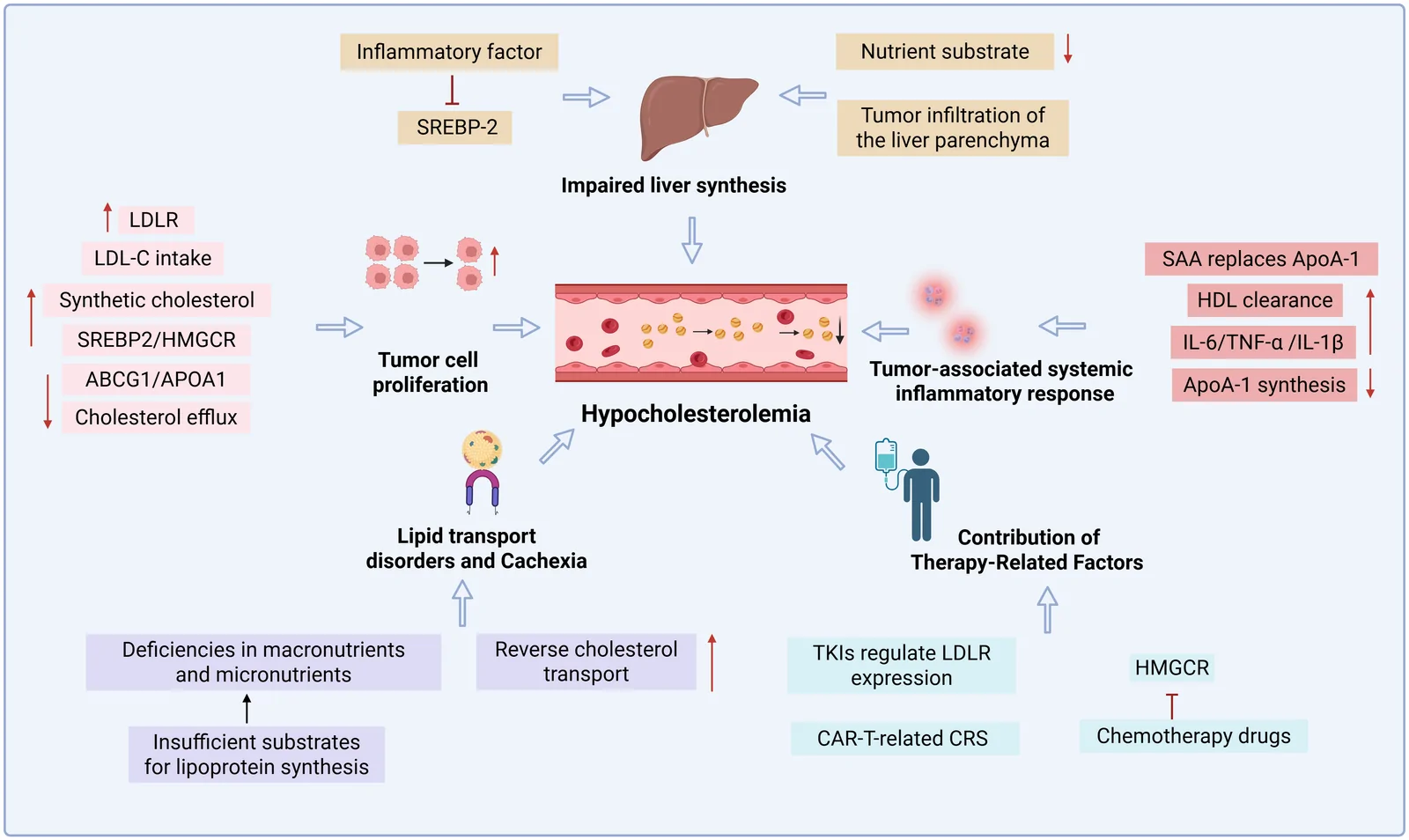
The mechanisms underlying tumor-associated hypocholesterolemia (Created in BioRender). (1) Predatory cholesterol consumption by malignant hematologic tumor cells via up-regulated SREBP-2-LDLR-mediated uptake and biosynthesis; (2) Impaired hepatic cholesterol synthesis caused by tumor infiltration and inflammatory cytokines suppressing SREBP-2; (3) Lipid-transport disturbance, malnutrition and cancer cachexia reducing lipoprotein substrate supply; (4)Tumor-induced systemic inflammatory responses (IL-6, TNF-ɑ, IL-1β) lead to accelerated HDL clearance and reduced availability of substrates for lipoprotein synthesis by inhibiting ApoA-I synthesis, promoting the replacement of ApoA-I by SAA, and impairing hepatic synthetic function; (5) Treatment-related factors may also contribute to the development of hypocholesterolemia.

### 4.1 The “predatory” metabolic demands of tumor cells

Cholesterol is indispensable for the rapid proliferation of cancer cells and functions at multiple biological levels. As an essential structural component of the phospholipid bilayer, cholesterol maintains membrane stability and regulates membrane fluidity, which is critical for sustained cell division and membrane biosynthesis (51, 87). In addition, cholesterol provides a platform for cellular signalling through the formation of lipid rafts and microdomains, which serve as key regulators of cell fate (88). Consequently, the homeostasis of cholesterol synthesis, uptake, storage, and efflux profoundly influences tumor growth.

To meet their unrestrained proliferative demands, tumor cells have evolved efficient cholesterol management strategies. On the one hand, they increase cholesterol uptake and synthesis. For example, by upregulating the expression of the LDLR, tumor cells markedly enhance their capacity to internalise LDL-C from the circulation (89, 90). Simultaneously, the expression of 3-hydroxy-3-methylglutaryl-CoA reductase (HMGCR), the rate-limiting enzyme in the cholesterol biosynthesis pathway, is upregulated in tumor cells, thereby promoting endogenous cholesterol production to support rapid proliferation (52). On the other hand, tumor cells reduce cholesterol efflux to prevent its loss.

Metabolic reprogramming is particularly prominent in DLBCL. Cellular studies have shown that DLBCL cell lines exhibit significantly increased expression of proteins and enzymes that promote cholesterol uptake and synthesis, including CD36, SREBP2, and HMGCR. Conversely, the expression of proteins that facilitate cholesterol efflux, such as NR1H2, ApoA-I), and ABCG1, is markedly reduced. This dual effect—enhanced uptake and synthesis coupled with diminished efflux—collectively drives the malignant proliferation of DLBCL cells (51). Notably, intrinsic metabolic adjustments are not observed. In various solid tumors and hematological malignancies, the SREBP2 pathway, a master transcriptional regulator of cholesterol synthesis, is persistently activated. This activation drives increased flux through the mevalonate pathway to meet the substantial demand for membrane components and signalling molecules (52).The combination of enhanced endogenous synthesis (driven by SREBP2/HMGCR) and augmented exogenous uptake (mediated by LDLR/CD36) constitutes a “dual-assurance” mechanism, ensuring that tumor cells maintain an adequate cholesterol supply even under conditions of reduced systemic cholesterol availability.

Collectively, these processes result in the extraction and sequestration of large amounts of plasma cholesterol within tumors, ultimately manifesting clinically as hypocholesterolemia.

### 4.2 Dysfunctional hepatic synthesis

The liver serves as the central organ for systemic cholesterol synthesis and metabolism (91). Hematological malignancies can impair hepatic function through multiple mechanisms, leading to reduced cholesterol synthesis. First, tumor cells may directly infiltrate the hepatic parenchyma, disrupting hepatocellular architecture and function, thereby inhibiting the activity of key cholesterol synthesis enzymes (e.g., HMG-CoA reductase and squalene epoxidase) and reducing hepatic cholesterol synthesis efficiency (92, 93). Second, tumor-associated inflammatory responses may induce a chronic inflammatory state within the liver. Inflammatory cytokines (such as IL-1 and IL-6) interfere with hepatic lipid metabolism by suppressing the activation of SREBP-2, thereby downregulating cholesterol synthesis (94). In addition, symptoms commonly observed in patients, including nausea, vomiting, and anorexia, lead to inadequate nutritional intake. This deficiency in essential substrates for cholesterol synthesis (e.g., acetyl-CoA and fatty acids) further compromises the liver’s capacity for cholesterol production (95).

### 4.3 Lipid transport dysfunction, malnutrition, and cachexia in cancer

One hypothesis proposes that macrophages and other immune cells within the tumor microenvironment become activated, thereby enhancing reverse cholesterol transport to the liver and contributing to reduced circulating cholesterol levels. However, the specific role and significance of this mechanism in hematological malignancies require further experimental validation (96).

Patients with advanced disease frequently present with anorexia, malabsorption, and cachexia, leading to insufficient intake of both macronutrients and micronutrients. This directly compromises substrate availability for lipoprotein synthesis, thereby exacerbating hypocholesterolemia. Systemic metabolic disturbances associated with cachexia further amplify abnormalities in lipid metabolism (97).

### 4.4 Tumor-associated systemic inflammatory response

Patients with hematological malignancies frequently exhibit chronic inflammation. Inflammatory cells, as well as tumors and their microenvironment, secrete elevated levels of cytokines, such as TNF-α, IL-6, and IL-1β, which modulate the expression of genes involved in hepatic lipid metabolism. Systemically, these cytokines act on the liver to suppress the synthesis of ApoA-I, the principal protein component of HDL, while inducing the expression of acute-phase proteins, such as serum amyloid A (SAA). The incorporation of SAA into HDL particles displaces ApoA-I, leading to structural abnormalities, functional impairment, and accelerated clearance of HDL. These changes ultimately result in significant hypocholesterolemia (94).

Accumulating evidence implicates abnormal lipid metabolism in the pathogenesis of various cancers (98, 99). Importantly, such metabolic dysregulation influences both inflammatory responses and immune regulation, which are critical drivers of tumor initiation and progression (100).

### 4.5 Contribution of therapy-related factors

Chemotherapeutic agents can disrupt cholesterol homeostasis through diverse mechanisms. For example, anthracyclines (such as doxorubicin) have been shown to suppress the activity of HMGCR, the rate-limiting enzyme in cholesterol synthesis, and to impair ABCA1-mediated cholesterol efflux via inhibition of LXR-α and PPAR-γ (101). In contrast, alkylating agents such as cyclophosphamide do not appear to directly affect hepatic lipogenesis and may even be associated with improved lipid profiles when used without anthracyclines (38). The effects of purine analogues on hepatic cholesterol synthesis remain unclear, although other antimetabolites such as methotrexate have been linked to alterations in ABCA1 and 27-hydroxylase expression (102, 103). These findings highlight the heterogeneity of the effects of chemotherapeutic agents on lipid metabolism and underscore the need for agent-specific considerations in managing cardiovascular risk in cancer survivors. Moreover, chemotherapy-induced gastrointestinal toxicity exacerbates malabsorption, further reducing cholesterol levels (104).

Clinical observations and mechanistic studies have shown that certain tyrosine kinase inhibitors (TKIs) can regulate serum cholesterol levels either by modulating the expression of lipid metabolism-related genes (e.g., LDLR and Apobec1) or by inhibiting the PDGFR signalling pathway, thereby affecting lipoprotein metabolism (105). Among these, imatinib has been associated with mild to moderate hypocholesterolemia in some patients (105), whereas nilotinib exhibits the opposite effect and tends to induce hypercholesterolemia (106).

During CAR-T cell therapy, serum total cholesterol, HDL-C, and LDL-C levels typically reach a nadir between days 8 and 14 post-infusion, correlating with the severity of cytokine release syndrome (CRS). Systemic inflammation and metabolic dysregulation driven by CRS have been proposed as key mechanisms, with cholesterol levels recovering upon clinical resolution (107).

## 5 The impact of hypocholesterolemia on hematologic malignancies

### 5.1 Promote tumor cell proliferation and enhance invasiveness

Cholesterol is a fundamental component of cellular membranes, including the plasma membrane, and an adequate supply is essential for cell proliferation (108). Tumor cells take up large amounts of circulating cholesterol, leading to hypocholesterolemia. Following uptake, cholesterol promotes tumor cell proliferation by upregulating the expression of key proliferation-associated proteins (109).Notably, tumor proliferation further accelerates cholesterol consumption, thereby creating a mutually reinforcing vicious cycle between hypocholesterolemia and tumor progression. At the same time, a sustained low-cholesterol environment exerts strong selective pressure on tumor cell populations. Subclones capable of upregulating low-density lipoprotein receptor expression, enhancing *de novo* cholesterol synthesis, and utilising cholesterol more efficiently gain a significant growth advantage. Consequently, these aggressive subpopulations are preferentially selected, potentially driving increased tumor invasiveness (110) (**Figure 4A**). Furthermore, cholesterol deprivation may disrupt the activation of certain oncogenic signalling pathways (e.g., PI3K/Akt) within lipid rafts (34, 35), thereby compelling tumor cells to activate alternative survival signalling cascades and ultimately acquire resistance to conventional chemotherapy.

**Figure 4:**
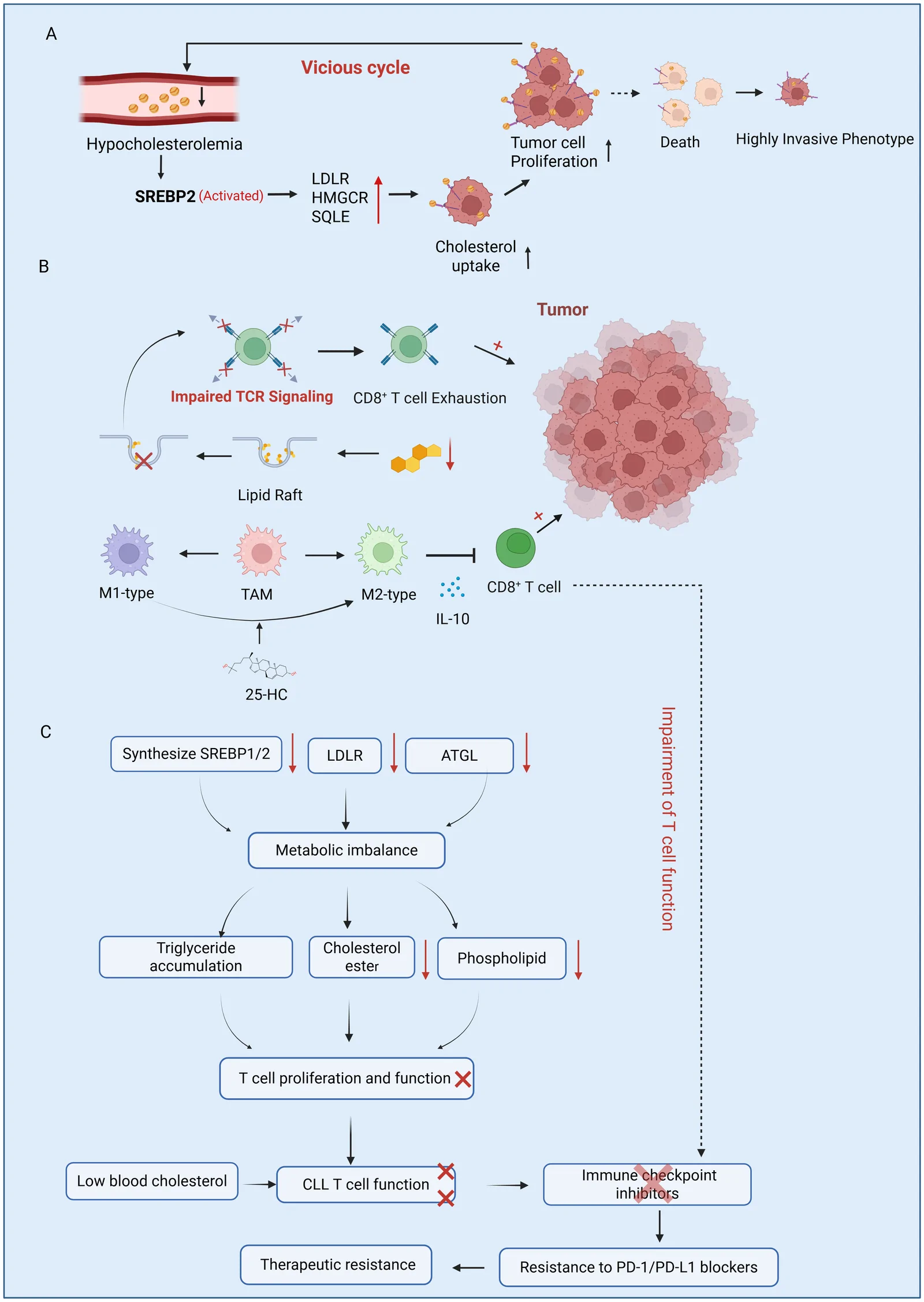
The impact of hypocholesterolemia on hematologic malignancies (Created in BioRender). Hypocholesterolemia promotes tumor cell proliferation and invasiveness through activation of the SREBP2-LDLR-HMGCR-SQLE axis **(A)**, and reshapes the immune microenvironment by impairing CD8^+^ T-cell function and promoting M2-like macrophage polarization via 25-HC **(B)**. In CLL, impaired cholesterol metabolism (SREBP1/2 ↓, LDLR ↓, ATGL deficiency) compromises T-cell proliferation and contributes to resistance to PD-1/PD-L1 blockade **(C)**. Additional mechanisms not depicted are discussed in the main text.

### 5.2 Reprogram the immune microenvironment

The activation, clonal proliferation, and effector functions of T cells are critically dependent on the integrity of plasma membrane lipid rafts (38, 39). A low-cholesterol environment, particularly reduced HDL levels, disrupts lipid raft structure, impairs T-cell receptor (TCR) signal transduction, and leads to T-cell dysfunction or exhaustion. Studies have shown that exogenous supplementation with cholesterol or HDL mimetics can restore the antitumor activity of CD8^+^ T cells (40). Moreover, HDL and its associated apolipoproteins can modulate the maturation, migration, and cytokine secretion profiles of antigen-presenting cells (such as dendritic cells) through interactions with cell surface receptors (e.g., SR-BI), thereby influencing the activation of naïve T cells. Reduced HDL-C levels may impair the initiation of antitumor immune responses (111). Collectively, these effects drive the Tumor Microenvironment (TME) towards an immunosuppressive state, facilitating tumor immune evasion (112).

Macrophages can polarise into two distinct phenotypes depending on the stimulus: M1 (pro-inflammatory) or M2 (anti-inflammatory) (55). M2 macrophages can promote tumor cell migration, invasion, and distant metastasis (113, 114). Altered expression of the cholesterol metabolite 25-hydroxycholesterol (25-HC) under hypocholesterolemic conditions can promote the polarization of tumor-associated macrophages (TAMs) towards an immunosuppressive M2-like phenotype. These M2-like TAMs secrete cytokines, such as IL-10, thereby suppressing effector T-cell function (56) (**Figure 4B**). Patients with AML and poor prognosis have been shown to exhibit an increased frequency of M2 macrophages (115).

Tumor-associated hypocholesterolemia reshapes the metabolic microenvironment by driving malignant cells to competitively consume circulating cholesterol, which alters cholesterol accessibility for multiple immune cell subsets beyond T cells and macrophages. Insufficient cholesterol bioavailability significantly impairs the cytotoxic effector function of NK cells. Tumor-secreted oxysterols including 25-hydroxycholesterol and 27-hydroxycholesterol further suppress SREBP-dependent metabolic reprogramming of NK cells and weaken their antitumor activity (116). Deficient cholesterol supply also disrupts the migration and antigen presentation capability of dendritic cells. LXR-mediated cholesterol efflux dynamically modulates the expression of MHC-II molecules and co-stimulatory signals to further weaken anti-tumor immune activation. Altered cholesterol metabolism enhances ACAT-dependent cholesterol esterification within regulatory T cells, which stabilizes their immunosuppressive phenotype under hypocholesterolemic conditions. Accumulated oxidized cholesterol metabolites additionally promote the recruitment and activation of MDSCs, ultimately facilitating the formation of an immunosuppressive niche that accelerates hematological malignancy progression.

### 5.3 Elevate the potential for therapy resistance

The cholesterol content of the cell membrane directly influences drug fluidity and permeability. Several studies have shown that modulation of membrane cholesterol levels can affect the intracellular accumulation of chemotherapeutic agents, such as anthracyclines, thereby altering tumor cell sensitivity to these drugs (117). In patients with CLL, T cells exhibit multi-level impairments in cholesterol metabolism, including reduced synthesis (decreased expression of SREBP1/2 and FASN), impaired uptake (downregulated LDLR), and defective utilisation (ATGL deficiency and reduced lipid oxidation). These disruptions lead to intracellular triglyceride accumulation, accompanied by deficiencies in cholesteryl esters and phospholipids. This metabolic imbalance directly compromises T-cell proliferation and represents a key mechanism underlying T-cell dysfunction in CLL (53). In the context of hypocholesterolemia, the already reduced exogenous cholesterol supply to T cells in patients with CLL may be further diminished, thereby potentially exacerbating functional impairment and compromising the efficacy of T cell-dependent immunotherapies. T-cell dysfunction, together with an immunosuppressive tumor microenvironment, directly undermines the effectiveness of immune checkpoint inhibitors, such as PD-1/PD-L1 blockade (**Figure 4C**). This mechanism contributes significantly to the lack of response observed in a subset of patients receiving immunotherapy (54).

Furthermore, cholesterol metabolic reprogramming also drives resistance to conventional chemotherapy and targeted agents across hematological malignancies. In AML, disrupted cholesterol homeostasis confers cytarabine resistance mainly through the SREBF1-ACSF2 axis and STAT3-SREBP1-mediated lipid remodeling, which enhances mitochondrial cholesterol esterification and prosurvival signaling (118, 119). In CML, BCR-ABL-driven cholesteryl-ester accumulation contributes to imatinib resistance independent of BCR-ABL kinase-domain mutations; ACAT-1 inhibition by avasimibe can reverse this phenotype via MAPK suppression (120). In B-cell-derived neoplasms including CLL, DLBCL and MM, upregulated cholesterol synthesis and uptake (SREBP, HMGCR, LDLR, SR-BI) maintain lipid-raft integrity and sustain oncogenic B-cell signaling, mediating resistance against BTKi, EZH2-inhibitors and bortezomib. Tumor-associated macrophages further shield myeloma cells from bortezomib-induced cytotoxicity via SREBP1-dependent lipid reprogramming (121–123).

Collectively, cholesterol metabolism dysregulation triggers distinct molecular alterations and functional outcomes across individual hematological neoplasms. The subtype-specific regulatory nodes and corresponding biological consequences are summarized in **Figure 5**.

**Figure 5:**
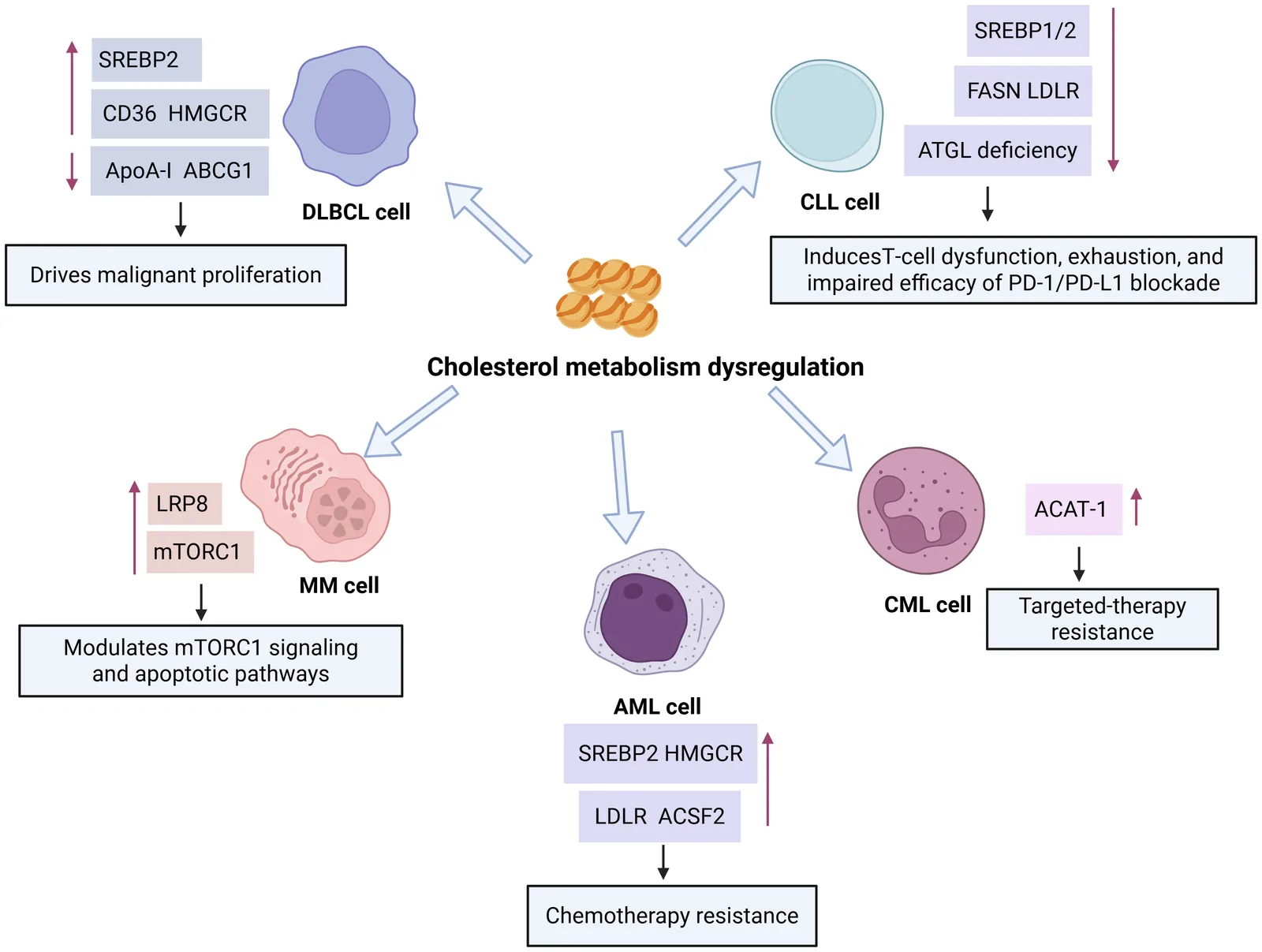
Subtype-specific effects of cholesterol-metabolism dysregulation (Created in BioRender). Dysregulated cholesterol-related molecules vary among DLBCL, CLL, MM, AML and CML cells, leading to diverse biological consequences including malignant proliferation, T-cell dysfunction, chemotherapy resistance and targeted-therapy resistance.

## 6 Clinical significance in hematological malignancies

Cholesterol level testing, as a simple, cost-effective, and routine biochemical parameter, holds significant clinical value in the screening, diagnosis, therapeutic monitoring, prognostication, and treatment guidance of hematological malignancies. Its potential utility has been substantiated in multiple clinical studies.

### 6.1 Auxiliary indicators for diagnosis

Hypocholesterolemia represents a valuable and low-cost adjunctive diagnostic marker for the initial evaluation of suspected hematological malignancies. Large-scale prospective evidence, such as the UK Biobank cohort study involving over 500,000 participants, demonstrated that during a median follow-up of 14.2 years, 1,819 cases of plasma cell tumors occurred. Individuals with lower baseline levels of total cholesterol, low-density lipoprotein cholesterol, high-density lipoprotein cholesterol, apolipoprotein A, and apolipoprotein B had a significantly increased risk of developing plasma cell tumors (84). However, Mendelian randomisation analysis in this study confirmed that this association is not causal, as no genetic evidence linked lipid levels to the development of multiple myeloma. Conversely, hypocholesterolemia can be mechanistically explained by the increased metabolic demands of malignant cells: tumor cells upregulate low-density lipoprotein receptor activity to sequester host cholesterol for rapid proliferation, a phenomenon first described in acute leukemia (124). Additionally, in the differential diagnosis between myelodysplastic syndrome (MDS) and aplastic anemia, the former is often associated with hypocholesterolemia, whereas the latter typically exhibits normal lipid levels, providing a useful clinical reference (125).

Therefore, in clinical practice, unexplained persistent hypocholesterolemia, particularly when accompanied by cytopenia, elevated globulin levels, or bone pain, should prompt further diagnostic evaluation, including serum protein electrophoresis, immunofixation electrophoresis, and bone marrow examination. Compared with bone marrow biopsy (the gold-standard test), serum protein electrophoresis, LDH, β2-MG and flow cytometry, serum cholesterol assay is inexpensive for routine longitudinal monitoring, with alterations detectable several years in advance. Owing to insufficient specificity and inability for independent diagnosis, cholesterol is suitable as an auxiliary screening marker, and its combination with conventional indicators can improve early disease identification, particularly in resource-limited settings where advanced biomarker testing is unavailable.

### 6.2 Tool for treatment efficacy evaluation and disease monitoring

Changes in serum cholesterol levels have emerged as a useful tool for assessing treatment efficacy and monitoring disease activity, as these changes often precede improvements in blood counts or imaging findings, which are traditional indicators of recovery. In a large prospective cohort study involving 761 patients with chronic lymphocytic leukemia, those who achieved complete or partial remission following chemotherapy exhibited significant increases in total cholesterol, high-density lipoprotein cholesterol, and low-density lipoprotein cholesterol compared with baseline, and these increases were directly associated with favourable survival outcomes (126). This phenomenon is attributed to the cessation of tumor-mediated cholesterol consumption; effective treatment eliminates malignant clones, thereby reversing the “metabolic hijacking” of host lipids and allowing the liver to resume normal cholesterol synthesis. The temporal dynamics of this recovery are clinically relevant, as lipid normalisation may occur before the full recovery of blood cell counts, making it an early indicator of treatment response. Conversely, in patients in remission, a progressive and unexplained decline in cholesterol levels during follow-up may indicate impending relapse, potentially preceding detectable cytopenia or overt lymphocytosis. This clinical utility is further supported by studies in acute leukemia, in which serum cholesterol levels were negatively correlated with tumor burden and returned to normal following successful induction therapy (89). Therefore, continuous monitoring of the lipid profile at diagnosis, during treatment, and throughout long-term follow-up provides a simple, low-cost, and dynamic indicator of disease activity, enabling earlier identification of treatment failure or relapse.

### 6.3 Prognostic stratification and risk assessment biomarkers

In addition to their diagnostic and monitoring roles, low cholesterol levels have been identified as independent prognostic factors in various hematological malignancies, and their prognostic value may complement or optimise traditional risk stratification models. The most compelling recent evidence derives from a 2026 multicentre study involving 305 patients with myeloproliferative neoplasms: after adjustment for potential confounders, patients with total cholesterol levels below 120 mg/dL had a threefold increased risk of disease progression (including transformation to myelofibrosis or acute leukemia) and an 87% increased risk of all-cause mortality compared with those with total cholesterol ≥150 mg/dL (127). Similarly, in chronic lymphocytic leukemia, lower baseline levels of total cholesterol, low-density lipoprotein cholesterol, and high-density lipoprotein cholesterol were independently associated with a shorter time to first treatment and reduced cancer-specific survival. Notably, the combination of low HDL-C and low LDL-C levels was a particularly strong predictor, with hazard ratios of 1.488 and 2.907 for time to first treatment and cancer-specific survival, respectively (126). Moreover, low LDL-C levels in patients with acute promyelocytic leukemia are associated with poor prognosis and may serve as a supplementary indicator for risk stratification (69). Based on these findings, an improved prognostic model, “ModelLipo-IPI,” was developed by integrating lipid parameters into the existing International Prognostic Index for chronic lymphocytic leukemia, demonstrating superior discriminatory ability and net clinical benefit in predicting survival outcomes. Similar associations have been reported in peripheral T-cell lymphoma, where low levels of HDL-C and LDL-C are associated with poorer overall survival. Collectively, these findings support the inclusion of baseline and serial lipid profiles in routine prognostic assessment to identify high-risk metabolic phenotypes. Such patients may require closer follow-up and potentially more intensive treatment strategies.

Compared with conventional risk-stratified systems including IPI, CLL-IPI, NCCN-IPI, ISS/R-ISS for MM and molecular genetic markers such as TP53 mutation, serum lipid profiles are simple and repeatable to dynamically reflect disease activity. They may complement existing prognostic models for better risk discrimination, yet cannot replace genetic tests given multiple confounding factors.

### 6.4 Optimization of treatment strategies and development of novel targets

Cholesterol metabolic pathways serve not only as biomarkers but also as promising therapeutic targets. Existing lipid-lowering agents have demonstrated potential antitumor activity in preclinical and early clinical studies. Statins, which inhibit HMG-CoA reductase, have attracted particular attention due to their ability to block the mevalonate pathway, which is essential not only for cholesterol synthesis but also for the post-translational isoprenylation of key oncoproteins such as Ras and Rho. Atorvastatin (80 mg/day) monotherapy was evaluated in patients with low-grade or refractory non-Hodgkin lymphoma (NCT00185731), based on the hypothesis that HMG-CoA reductase inhibition downregulates lymphoma-associated oncogenes. While full trial results remain unpublished, a 2024 study confirmed that atorvastatin significantly alters the expression profile of oncogenic miRNAs in NHL plasma (128). Pitavastatin added to venetoclax-based therapy in CLL/SLL and AML demonstrated complete remission in all evaluable patients; one CLL patient showed a rapid leukemic cell reduction from 38% to 4% after a single pitavastatin dose (NCT04512105) (129). A retrospective study of patients with CML under watchful waiting reported that initiation of statin therapy was associated with accelerated lymphocytosis and a shorter time to first treatment, suggesting that the effects of statins may vary according to disease stage, statin type, or patient subgroup (130). In addition to statins, liver X receptor (LXR) α/β agonists exert anti-proliferative and immunomodulatory effects across AML, ALL and MM pre-clinical models (131, 132). Preclinical studies have shown that avasimibe (an ACAT-1 inhibitor) in combination with imatinib exhibits synergistic antiproliferative effects in drug-resistant CML models (cell-based and mouse) lacking the T315I mutation; this mechanism is associated with the downregulation of the MAPK pathway and the reversal of cholesterol ester accumulation. Although this combination is ineffective against the T315I mutation, it targets the majority (50–85%) of treatment-resistant patients without kinase mutations, which is of significant clinical importance (120). Moreover, inhibition of PCSK9 stabilises major histocompatibility complex class I (MHC class I) molecules on the surface of tumor cells, thereby enhancing T-cell recognition and potentially producing synergistic effects with immune checkpoint inhibitors (133, 134). While PCSK9 inhibitors have demonstrated immunomodulatory and antitumor effects in preclinical models of solid tumors, their application in hematological malignancies remains to be formally investigated, and no dedicated preclinical or clinical studies have been reported to date (**Table 2**). Although the biological rationale for targeting cholesterol metabolism in hematological malignancies is strong, the routine use of these agents for antitumor purposes lacks high-level evidence from prospective randomised controlled trials. Their clinical application remains largely confined to the management of treatment-related dyslipidemia or within the context of well-designed clinical trials, highlighting important directions for future research.

**Table 2:** Agents targeting cholesterol metabolism in hematological malignancies.

Agent	Treatment	Target	Application	Experiment status	Clinical trial
Atorvastatin	Add-on to anthracycline-based chemotherapy	HMGCR	Lymphoma	Phase II; Completed	NCT02943590
Pitavastatin	Add-on to venetoclax-based therapy	HMGCR	AML, CLL/SLL	Phase I; Terminated	NCT04512105
PCSK9 inhibitors	Monoclonal antibodies	PCSK9	Solid tumors only; none in hematologic	//	//
LXR agonists	Monotherapy	LXRα/β	AML, ALL, MM	Preclinical	//
Avasimibe	Combine with imatinib	ACAT-1	CML (imatinib-resistant)	Preclinical	//

## 7 Conclusions and future prospects

Dysregulated cholesterol homeostasis plays a complex and pivotal role in the development and progression of hematological malignancies. It not only directly influences disease progression by regulating tumor cell proliferation but also promotes tumor growth and survival by reshaping the bone marrow microenvironment. Cholesterol, as a simple and cost-effective routine biochemical parameter, has demonstrated substantial clinical value in screening, efficacy assessment, prognostic evaluation, and treatment guidance in hematological malignancies. Hypocholesterolemia represents not only a hallmark of tumor-associated metabolic hijacking but also an independent indicator of poor prognosis. In recent years, significant progress has been made in therapies targeting cholesterol metabolism, representing a promising new direction for treatment. Multiple small-molecule agents targeting cholesterol metabolic pathways, as well as combination therapeutic strategies, have demonstrated potential in the treatment of hematological malignancies. However, several challenges remain. These include the complexity of the mechanisms underlying dysregulated cholesterol metabolism across different hematological tumor subtypes, the need to further improve the safety and efficacy of targeted therapies, and the lack of specific biomarkers for precise diagnosis and prognostic assessment. As the mechanisms linking cholesterol metabolism dysregulation to hematological malignancy progression continue to be elucidated, and as novel targeted agents and combination strategies are developed, therapeutic approaches based on cholesterol metabolism modulation are expected to play an increasingly important role in clinical management. These holds promise for improving patient outcomes and survival.
